# Vaginal microbiota and human papillomavirus infection among young Swedish women

**DOI:** 10.1038/s41522-020-00146-8

**Published:** 2020-10-12

**Authors:** Liqin Cheng, Johanna Norenhag, Yue O. O. Hu, Nele Brusselaers, Emma Fransson, Andreas Ährlund-Richter, Unnur Guðnadóttir, Pia Angelidou, Yinghua Zha, Marica Hamsten, Ina Schuppe-Koistinen, Matts Olovsson, Lars Engstrand, Juan Du

**Affiliations:** 1 Department of Microbiology, Tumor and Cell Biology, Centre for Translational Microbiome Research (CTMR), Karolinska Institutet, Stockholm, Sweden; 2 Department of Women’s and Children’s Health, Uppsala University, Uppsala, Sweden; 3 Department of Oncology and Pathology, Karolinska Institutet, Stockholm, Sweden; 4 Science for Life Laboratory, Karolinska Institutet, Stockholm, Sweden

**Keywords:** Microbiome, Infectious-disease diagnostics

## Abstract

Human papillomavirus (HPV) infection is one of the most common sexually transmitted diseases. To define the HPV-associated microbial community among a high vaccination coverage population, we carried out a cross-sectional study with 345 young Swedish women. The microbial composition and its association with HPV infection, including 27 HPV types, were analyzed. Microbial alpha-diversity was found significantly higher in the HPV-infected group (especially with oncogenic HPV types and multiple HPV types), compared with the HPV negative group. The vaginal microbiota among HPV-infected women was characterized by a larger number of bacterial vaginosis-associated bacteria (BVAB), *Sneathia*, *Prevotella*, and *Megasphaera*. In addition, the correlation analysis demonstrated that twice as many women with non-*Lactobacillus-*dominant vaginal microbiota were infected with oncogenic HPV types, compared with *L. crispatus-*dominated vaginal microbiota. The data suggest that HPV infection, especially oncogenic HPV types, is strongly associated with a non-*Lactobacillus-*dominant vaginal microbiota, regardless of age and vaccination status.

## Introduction

Infection with human papillomavirus (HPV) is among the most common sexually transmitted diseases in the world, with the highest prevalence among women below 25^1,2^. HPV infection is the main cause of cervical cancer and is related to many other cancers, including head and neck cancer^3^. Depending on their oncogenic potential, mucosal HPV types can be divided into oncogenic HPVs, such as those observed in cancer cases, and non-oncogenic HPVs, mainly found in condyloma^4^. The two most common HPV types in cervical cancer are HPV16 and 18, which are responsible for ~70% of cervical cancer cases worldwide^5,6^. At a youth clinic in Stockholm, Sweden, we have previously shown an overall cervical HPV prevalence of over 70% among young girls in Sweden^7–9^. The HPV vaccination program was gradually introduced to Sweden from 2007. Since 2012, all girls between the ages of 10 and 12 years are offered free vaccination with the quadrivalent Gardasil vaccine against HPV6, 11, 16, and 18, in a school-based vaccination program and catch-up vaccination. The vaccination ratio has increased dramatically from 10.7% (2008–2010) to 82.1% (2017–2018)^7^. The prevalence of HPV types covered in the vaccine has dropped significantly in vaccinated women compared with non-vaccinated women, underlining the importance and success of the vaccination program^7,8^. However, the total HPV prevalence caused by HPV types that are not covered by the vaccine is still high, indicating that more interventions to reduce these HPV infections are still needed^7,8^. Further, the influence of the HPV vaccine on vaginal microbiota has not been thoroughly investigated, especially in a high vaccination coverage country.

An increasing number of studies suggest that vaginal microbiota play an essential role in women’s health, specifically in sexually transmitted diseases, pelvic inflammatory disease, and adverse obstetric outcomes^10–13^. The vaginal microbiota is primarily dominated by one of the four most common *Lactobacillus* species: *Lactobacillus crispatus*, *Lactobacillus iners, Lactobacillus gasseri*, and *Lactobacillus jensenii*^14–16^. In addition, some women may have vaginal microbiota dominated by bacterial species other than *Lactobacilli*, such as *Prevotella*, *Gardnerella*, and *Sneathia*^11,17,18^. The general clinical diagnostic approaches worldwide for bacterial vaginosis (BV), which is also characterized by a lack of *Lactobacilli* but a higher quantity of aerobic and anaerobic bacteria, are the Amsel criteria and the Nugent score, based on wet smear diagnosis and Gram staining. However, the sensitivity and specificity for both methods are moderate^19^. Molecular diagnosis, such as 16S rRNA gene sequencing, enables the microbiota determination at the species level. Bacterial vaginosis associated bacteria (BVAB), including BVAB 1, 2, and 3, have been identified from the vaginal fluid of women with bacterial vaginosis and could serve as potential vaginosis biomarkers^20–22^. Unfortunately, BVAB have not been included in the 16S amplicon sequencing-based vaginal microbiota studies related to HPV, probably due to taxonomic information missing from the popular 16S rRNA databases, with most studies on BVAB being based on qPCR sequencing.

Cross-sectional studies and very few longitudinal studies from other countries showed that *L. crispatus* is observed more frequently in women without HPV infection and cancer lesions, whereas *L. iners* and non-*Lactobacillus* species are more common in HPV-infected women and patients with cancer lesions^23–26^. However, there are no data available yet in the Nordic countries on the vaginal microbiota composition and its relationship with HPV infection, using a sequencing method. Thus, we initiated a cross-sectional study to assess the association between the vaginal microbiota and 27 HPV types in Sweden. In addition, because HPV infection has the highest prevalence among young women, we designed our study to focus on women below 30 years old.

## Results

### Participant characteristics in the study cohort

As seen from the flowchart of Supplementary Fig. 1, a total of 345 participants were enrolled in this study, in which 33 women were excluded from the study due to antibiotics usage within the past three months (*n* = 18) or incomplete clinical information (*n* = 15). Samples with low DNA concentration and low reads in sequencing (*n* = 55) were excluded from downstream microbiota analysis. Eventually, 169 samples from women visiting the youth clinic and 88 samples from women attending the cervical screening were included for analyses (Supplementary Fig. 1).

**Fig. 1:**
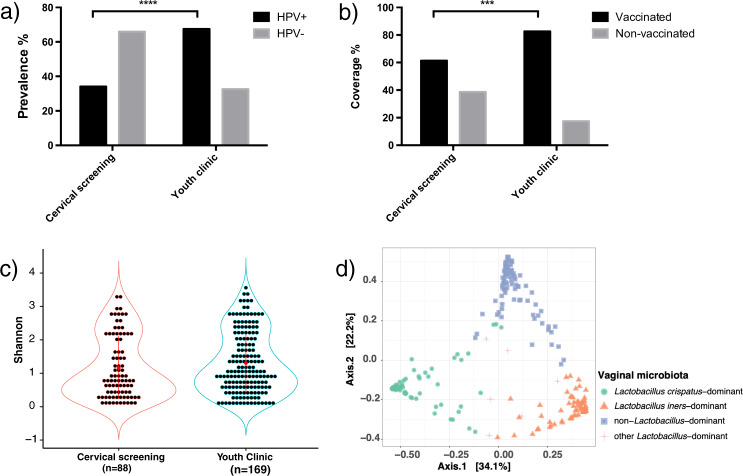
Comparison of HPV prevalence, HPV vaccination status, and microbial diversity in the youth clinic and the cervical screening samples. **a** Significantly higher HPV prevalence was observed from the youth clinic samples than the cervical screening samples. **b** Significantly higher HPV vaccination coverage was shown in samples from the youth clinic than samples from the cervical screening. **c** Microbial alpha diversity based on Shannon analysis did not show the difference between samples from the youth clinic and the cervical screening. Every dot in the violin plot represents one individual. Data were presented as mean values with standard deviations. **d** Principal coordinates analysis (PCoA) of microbial species data based on Bray-Curtis distance matrix demonstrated three main vaginal microbiota clusters. Statistical significance between the groups was tested by Fisher’s exact test in **a** and **b**, and by Wilcoxon rank-sum one-sided test in **c** (*p* = 0.108). ***p < 0.001 and ****p < 0.0001. HPV+: HPV-infected, HPV−: HPV-uninfected.

The HPV prevalence of the samples from the youth clinic have been published previously^7^. From the 169 women visiting the youth clinic, the prevalence of any HPV and oncogenic HPV were 67.5% and 59.8%, respectively (Supplementary Table 1). Among the 88 participants attending the cervical screening, the overall HPV prevalence was 34.1%, with HPV56 (6.8%), HPV45 (4.5%), and HPV52 (4.5%) as the three most common oncogenic HPV types (Supplementary Fig. 2a and Supplementary Table 1). The HPV vaccine appeared to provide full protection to the participants in the cervical screening, with no one (including the non-vaccinated ones) being infected with the HPV types covered in the quadrivalent HPV vaccine (Supplementary Fig. 2a, b). Moreover, when compared vaccinated with non-vaccinated women, none of the prevalence difference (any HPV, oncogenic HPVs, HPV covered in vaccine, probably oncogenic HPVs, and non-oncogenic HPVs) reached statistical significance according to Fisher’s exact test (Supplementary Fig. 2b). Both the HPV infection prevalence and vaccine coverage in the cervical screening samples were significantly lower than that of the youth clinic samples (34.1% vs. 67.5%, *p* < 0.0001 and 61.4% vs. 81.1%, *p* < 0.001, respectively; Figs. 1a, b and Supplementary Table 1).

### Vaginal microbiota was comparable in women from the youth clinic and the cervical screening

We continued to compare the microbial community composition from the two sources. Alpha diversity analysis of the microbiota profile based on Shannon and Faith’s diversity suggested no significant difference between the two sources (*p* = 0.064 and 0.138, respectively), while Chao 1 analysis showed a significant difference between the two sources (*p* = 0.004; Fig. 1c and Supplementary Figs. 3a and 4a). The bacterial community profiles in the samples from the youth clinic and the cervical screening samples were comparable, with overall 32.7% *L. crispatus* dominated, 30.4% *L. iners* dominated, and 33.5% non-*Lactobacillus*-dominated (Supplementary Table 1). Furthermore, no significant difference was observed in the ratio of the four community types based on sample source (Supplementary Table 2). In order to address the relationship between vaginal microbiota and HPV infection, we combined the samples from the youth clinic and the cervical screening for all the following analysis.

Principal coordinates analysis (PCoA) based on the Bray-Curtis distance demonstrated that all the samples were mainly separated into *L. crispatus-*, *L. iners-*, and non-*Lactobacillus*-dominated categories, and only a few samples were dominated by other *Lactobacillus* species (Fig. 1d). Detail distributions of the amplicon sequence variants (ASVs) from the major genera and species including *Gardnerella, Prevotella, Sneathia*, and BVABs were shown in Supplementary Fig. 5a. Furthermore, we also performed the PCoA based on the UniFrac phylogenetic distance, which separated all the samples into *Lactobacillus*-dominated and non-*Lactobacillus*-dominated categories (Supplementary Fig. 6a). Since *Lactobacillus* species are phylogenetically close, *Lactobacillus*-dominated samples clustered together in phylogenetic distance-based PCoA (Supplementary Fig. 6a). The contributions of the ASVs from the major genera and species were presented in the separated PCoA panels of Supplementary Fig. 6b.

### Young women with HPV infection had higher vaginal microbial diversity

**Fig. 2:**
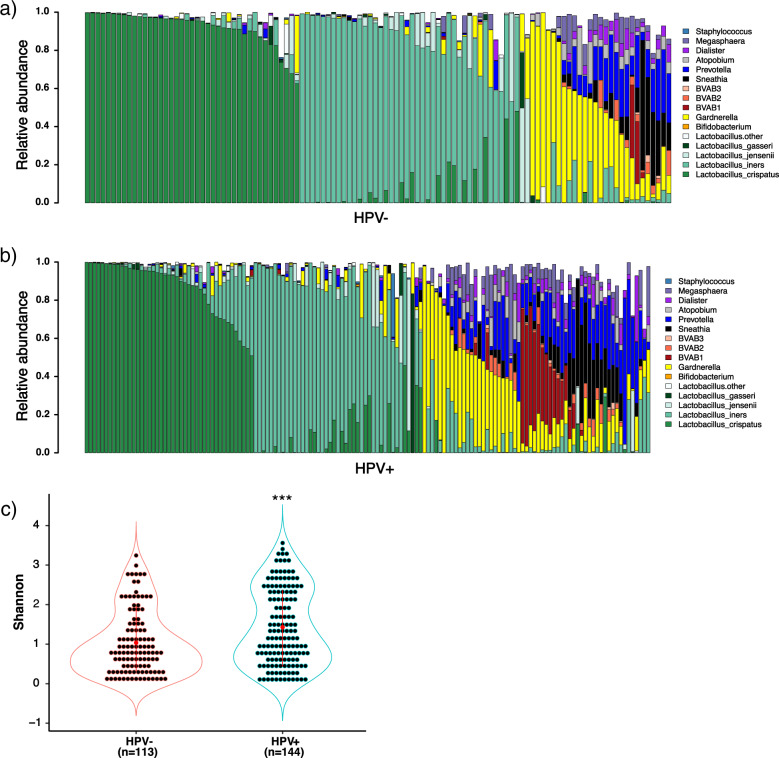
Difference in vaginal microbiota of HPV-uninfected and HPV-infected young women. **a** Vaginal microbiota at the genus/species level from HPV-uninfected young women. Except BVABs, the following criteria were used in order to show the important and abundant taxa clearly: (1) Bacteria with over 1% mean relative abundance in all the samples. (2) *Lactobacillus* species that have more than 10% of reads in any sample. (3) Non-*Lactobacillus* genera that have over 30% of reads in any sample. **b** Vaginal microbiota at genus/species level from HPV-infected young women. Same criteria were used as in **a**. **c** Microbial alpha diversity (Shannon) comparison between groups of HPV-uninfected and HPV-infected young women demonstrated a significantly higher vaginal microbiota diversity among HPV-infected women by Wilcoxon rank-sum one-sided test. Data were presented as mean values with standard deviations. ***p < 0.001. HPV+: HPV-infected, HPV−: HPV-uninfected.

In general, *L. crispatus* and *L. iners* were found to be the most dominant species among both HPV-uninfected and HPV-infected participants (Fig. 2a, b). The majority of non-*Lactobacillus*-dominated samples consisted of a large proportion of bacteria belonging to the genera *Gardnerella, Prevotella, Sneathia*, and BVABs (Fig. 2a, b). The Shannon, Chao 1, and Faith’s diversity analyses all found significantly higher microbial alpha diversity among HPV-infected women than those without HPV infection (*p* = 0.0006, 0.001, and 0.0005, respectively; Fig. 2c and Supplementary Figs. 3b and 4b). The ratios of the four vaginal microbiota compositions were significantly different between women with and without HPV infection (*p* = 0.043) (Supplementary Table 2). Notably, the non-*Lactobacillus* community profile was more prevalent among HPV-infected women, compared with uninfected women according to Fisher’s exact test (*p* = 0.011; Fig. 2a, b and Supplementary Table 2). However, the PCoA analysis based on the Bray–Curtis distance showed no clear separation between HPV-uninfected and HPV-infected groups based on their microbial community compositions (Supplementary Fig. 5b). The PCoA based on the UniFrac phylogenetic distance also did not separate the samples according to HPV infection status (Supplementary Fig. 6c).

### Young women with oncogenic HPV infection had higher vaginal microbial diversity

**Fig. 3:**
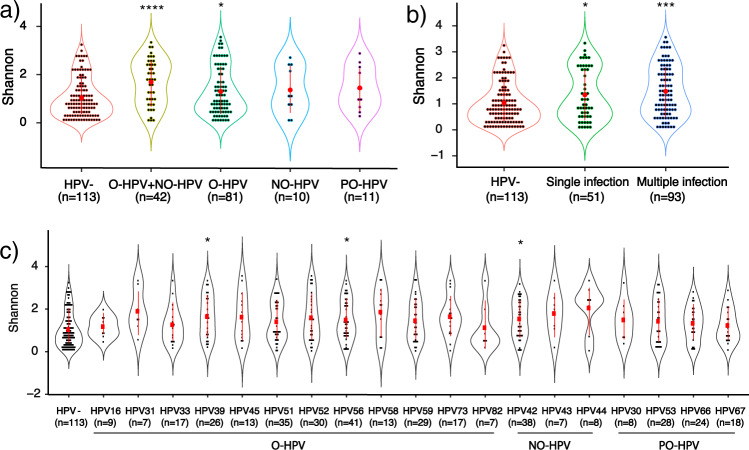
Microbial alpha diversity analysis based on Shannon index according to HPV oncogenic type, infected numbers and HPV types. **a** Microbiota diversity of participant group of uninfected women, and the groups infected with oncogenic plus non-oncogenic HPVs, oncogenic HPVs, non-oncogenic HPVs and probably oncogenic HPVs were compared. The five groups showed significantly different diversity (Kruskal–Wallis test; *p* < 0.05). Groups with oncogenic plus non-oncogenic HPVs and oncogenic HPVs showed statistical higher diversity compared with HPV-uninfected group (Wilcoxon one-side test; *p < 0.05 and ****p < 0.0001). **b** Microbiota diversity comparison of HPV-uninfected group, and groups infected with single and multiple HPV types. The diversities are significantly different among the three groups (Kruskal–Wallis test; *p* < 0.005). Significant higher microbiota diversity of participants infected with single and multiple HPV types was observed compared with HPV-uninfected women (Wilcoxon one-side test; *p < 0.05, ***p < 0.001). **c** Microbiota diversity among participants infected with different HPV types in comparison with uninfected women. Significantly higher microbiota diversity was observed with women infected with HPV39, 56, and 42, compared with uninfected women. Statistical significance between the groups was tested by Wilcoxon one-side test adjusted by Benjamini–Hochberg correction. (*q < 0.05). Data was presented as mean values with standard deviations. HPV−: HPV-uninfected, O-HPV: oncogenic HPV, NO-HPV: non-oncogenic HPV, PO-HPV: probably oncogenic HPV.

Microbial alpha diversity based on Shannon, Chao 1, and Faith’s diversity analyses displayed significantly higher diversity of women infected with both oncogenic and non-oncogenic HPVs, than HPV-uninfected women (*p* = 0.00008, 0.0002, and 0.0005, respectively; Fig. 3a and Supplementary Figs. 3c and 4c). The same holds true when comparing women with only oncogenic HPV infection with those without HPV infection (*p* = 0.041, 0.030, and 0.025, respectively; Fig. 3a and Supplementary Figs. 3c and 4c). Shannon and Faith’s diversity analyses also displayed significant differences between women infected with both oncogenic and non-oncogenic HPV, and women with only oncogenic HPV infection (*p* = 0.013 and 0.03, respectively), but no significant difference among other groups was observed. The PCoA analysis showed no clear separation of samples with HPV-uninfected, infected with both oncogenic and non-oncogenic HPVs, and only oncogenic HPVs (Supplementary Figs. 5c, d and 6d). We further divided samples according to their HPV phylogenetic groups and evaluated the association of HPVs phylogenetic groups with vaginal microbiome composition. However, PCoA based on the UniFrac phylogenetic distance showed no clear separation among samples from different HPV phylogenetic groups either (Supplementary Fig. 6e).

### Young women with multiple HPV types had higher vaginal microbial diversity

We further analyzed whether the number of infected HPV types affected vaginal microbial diversity. Compared with women without HPV infection, all the analyses showed that women infected with multiple HPV types had significantly higher microbiota diversity (*p* = 0.0004 for Shannon, 0.0007 for Chao 1, and 0.0008 for Faith’s diversity, respectively; Fig. 3b and Supplementary Figs. 3d and 4d). Only the Shannon analysis displayed significantly higher microbiota diversity in women infected with single HPV type than in women without HPV infection (*p* = 0.042; Fig. 3b and Supplementary Figs. 3d and 4d).

### Young women with certain HPV types had higher microbial diversity

Detailed information on 27 HPV types allowed us to compare the vaginal microbiome from participants infected with different HPV types. As listed in Fig. 3c, among the HPV types with enough women in the group for analysis (*n* > 5), women infected with HPV39, 42, and 56 had significantly higher diversity compared with HPV-uninfected group in the Shannon analysis, which indicates that infection by these three HPV types tends to be related to higher diversity in vaginal microbiota (Fig. 3c). HPV39 and 58 in the Chao 1 analysis and HPV39, 58, and 59 in the Faith’s diversity analysis were the HPV types showing significantly higher diversity compared with the HPV-uninfected group (Supplementary Figs. 3e and 4e).

### Certain bacterial species were related to HPV infection

**Fig. 4:**
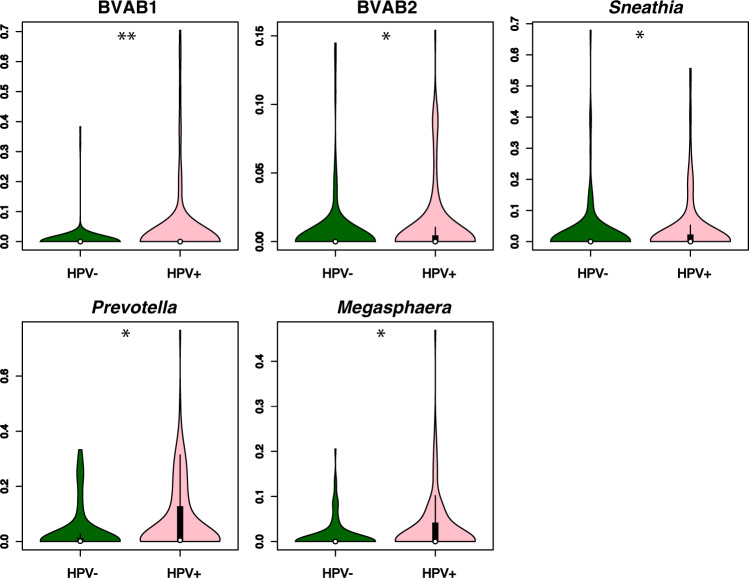
Bacterial species/genera presented significantly different in HPV-infected and HPV-uninfected women. BVAB1, BVAB2, *Sneathia*, *Prevotella*, and *Megasphaera* were the bacterial species/genera that significantly higher presented in HPV-infected women than HPV-uninfected women. Analysis only conducted on the taxa listed in Fig. 2. Statistical significance between the groups was tested by two-sided Wilcoxon rank-sum test adjusted by Benjamini–Hochberg correction. *q < 0.05, **q < 0.01. Data was presented as median values with the interquartile and upper adjacent values indicated by the thick and thin lines, separately. HPV+: HPV-infected, HPV−: HPV-uninfected.

To identify potential bacterial biomarkers for HPV infection, we compared the relative abundance of all the bacteria from women with and without HPV infection. From statistical analysis on microbiota taxonomy, we observed that BVAB 1, BVAB 2, *Sneathia*, *Prevotella*, and *Megasphaera* were significantly more prevalent among HPV-infected women than HPV-uninfected women (*q* = 0.0038, 0.048, 0.048, 0.048, and 0.048, respectively; Fig. 4). Interestingly, BVAB 1 almost exclusively presented in the vaginal microbiota of HPV-infected young women, indicating a very close relationship between BVAB 1 and HPV infection (Fig. 2a, 2b).

### Age and HPV vaccine had little influence on vaginal microbial diversity

**Fig. 5:**
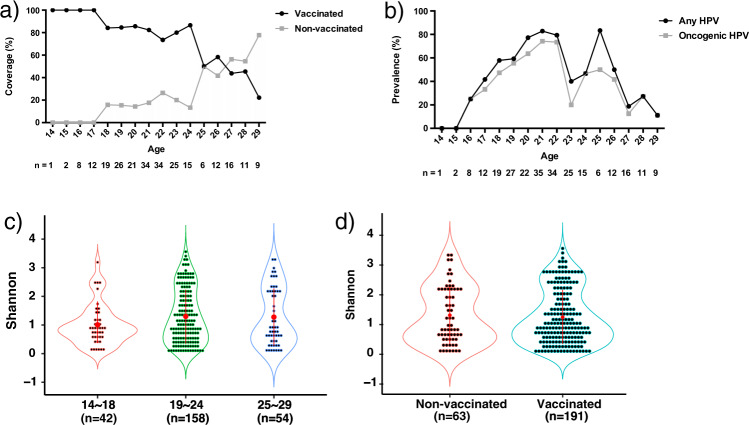
HPV infection and microbiota diversity based on HPV vaccination status and age. **a** HPV vaccination status in all the samples showed a decreased trend in vaccination coverage as age increased. **b** HPV prevalence and oncogenic HPV prevalence in all samples according to age. An overall high HPV prevalence was observed between age of 18–26. Most of the HPV infection contained oncogenic HPV in all the age groups. **c** Microbial alpha diversity based on Shannon index of all the samples showed no significant difference among age groups (Kruskal–Wallis test; *p* > 0.05). **d** Microbial alpha diversity based on Shannon index of all the samples did not show significant difference between the groups with and without vaccination (Wilcoxon rank-sum one-sided test; *p* > 0.05). Data were presented as mean values with standard deviations.

In general, HPV vaccine coverage declined with increasing age, probably due to the lag time of the national HPV vaccination program in Sweden. Vaccination coverage dropped from 100% among young women age 14–17 to 70–90% among those age 18–24, and 20–60% among those age 25–29 (Fig. 5a). Irrespective of vaccination status, HPV prevalence increased from age 14 (0%), and peaked around the age of 18-26 (~50% and above) and dropped to ~10% at age of 29 (Fig. 5b). Oncogenic HPV types accounted for most of the HPV-infected cases in each age group and followed similar trend as the total HPV infection (Fig. 5b)^7^.

However, the vaginal microbiota diversity analysis according to age showed no difference among the age groups in all three analysis (Fig. 5c and Supplementary Figs. 3f and 4f). Similarly, microbial alpha diversity of vaccinated women also showed no significant difference from non-vaccinated women (Fig. 5d and Supplementary Figs. 3g and 4g). In addition, these data were supported by no significant difference was shown in the ratio of the four community types among samples with different age and vaccine status (Supplementary Table 2). All together, these data suggested that age and HPV vaccine status had little influence on microbial composition.

### Age, vaccine status, and vaginal microbiota showed correlation to HPV infection

We continued to evaluate the correlation of age, vaccination status, and vaginal microbiota with the risk of HPV or oncogenic HPV infection. The significantly higher HPV prevalence in the youth clinic compared with the cervical screening samples, contributed to the highest ratio of any HPV, oncogenic HPV, or multi-type HPV infection observed among the 19–24 age group (Table 1 and Supplementary Table 3). HPV vaccination demonstrated a significant protective effect against multiple HPV infection (*p* < 0.001, Supplementary Table 3).

**Table 1:** The risk of human papillomavirus (HPV) by different characteristics.

	Unadjusted OR (95% CI)			Adjusted OR (95% CI)			Adjusted OR (95% CI)	
	HPV+	Oncogenic HPV	Non-oncogenic HPV	HPV+	Oncogenic HPV	Non-oncogenic HPV	Multiple HPV	Multiple oncogenic HPV
Age								
14–18 years	Reference	Reference	Reference	Reference	Reference	Reference	Reference	Reference
19–24 years	2.7 (1.4–5.4)^a^	2.5 (1.2–5.1)^a^	3.0 (1.1–8.2)^a^	4.0 (1.9-8.7)^a^	3.2 (1.5–6.8)^a^	3.3 (1.2–9.2)^a^	4.1 (1.7–9.7)^a^	3.7 (1.5–9.3)^a^
25–29 years	0.7 (0.3–1.5)	0.6 (0.3–1.5)	0.1 (0.0–1.2)	2.2 (0.6–7.6)	1.8 (0.5–6.4)	0.3 (0.0–3.1)	2.2 (0.4–12.4)	1.7 (0.3-11.1)
HPV vaccination								
No	Reference	Reference	Reference	Reference	Reference	Reference	Reference	Reference
Yes	1.0 (0.5–1.7)	0.9 (0.5–1.5)	1.0 (0.5–2.0)	0.5 (0.2–1.1)	0.5 (0.2–1.00)^a^	0.5 (0.2–1.0)	0.3 (0.1–0.9)^a^	0.4 (0.1–1.0)^a^
Sample source								
Screening cohort	Reference	Reference	Reference	Reference	Reference	Reference	Reference	Reference
Youth clinic	4.0 (2.3–6.9)^a^	4.5 (2.5–7.9)^a^	6.4 (2.4–16.8)^a^	5.4 (2.4–12.2)^a^	5.5 (2.4–12.6)^a^	3.7 (1.3–11.2)^a^	16.1 (4.9–53.3)^a^	17.4 (4.6–65.6)^a^
Vaginal microbiota								
*L. crispatus*	Reference	Reference	Reference	Reference	Reference	Reference	Reference	Reference
*L. iners*	0.9 (0.5–1.6)	1.3 (0.7–2.4)	1.00 (0.4–2.3)	0.7 (0.3–1.3)	1.0 (0.5–2.1)	0.8 (0.3–1.9)	0.8 (0.3–1.8)	1.0 (0.4–2.3)
Other *Lactobacilli*	1.9 (0.4–8.1)	0.4 (0.1–2.2)	1.6 (0.3–8.4)	2.1 (0.5–10.2)	0.4 (0.1–2.2)	1.4 (0.2–8.4)	2.2 (0.3–16.8)	0.7 (0.1–4.8)
Non-*Lactobacillus* dominated	2.0 (1.1–3.7)^a^	2.1 (1.2–4.0)^a^	2.2 (1.1–4.8)^a^	1.7 (0.9–3.5)	2.0 (1.0–3.9)^a^	2.0 (0.9–4.6)	2.5 (1.1–5.9)^a^	2.4 (1.0–5.8)^a^

All the data is presented as odds ratios (OR) and 95% confidence intervals (CI) calculated by multivariable logistic regression. The risk of (any subgroup of) HPV infection was compared to group of “HPV–” in all analyses (see raw number in supplementary Table 3).

^a^Statistically significant difference (<0.05).

Logistic regression analysis showed that age and the sample source were significantly associated with the risk of being HPV-infected. The highest HPV risks after adjustment were among women in the 19–24 age group (odds ratios: OR = 4.0, 95% confidence intervals: CI 1.9–8.7), compared with women below 18, and women from the youth clinic (OR = 5.4, 95% 2.4–12.2), compared with the cervical screening samples (Table 1). Similar associations were observed for both oncogenic HPV and non-oncogenic HPV types. After adjustment for the other variables, age (19–24 years old) and sample source (youth clinic), and non-*Lactobacillus* dominated remained significantly associated with infections with oncogenic HPV, multiple HPV, and multiple oncogenic HPV (Table 1 and Supplementary Table 3). Moreover, HPV vaccine halved the risk of oncogenic HPV infection (OR = 0.5, 95% CI 0.2–1.00) (Table 1 and Supplementary Table 3).

Non-*Lactobacillus-*dominated vaginal microbiota showed more than twice the risk of having an infection with any HPV, oncogenic HPV, and non-oncogenic HPV infection than those with *L. crispatus-*dominated microbiota (OR = 2.0, 95% CI 1.1–3.7 for HPV infection; OR = 2.1, 95% CI 1.2–4.0 for oncogenic HPV infection; and OR = 2.2, 95% CI 1.1–4.8 for non-oncogenic HPV infection). After adjustment for the other variables listed in the table, the risk of oncogenic HPV infection was OR = 2.0 (95% CI 1.0–3.9) in non-*Lactobacillus-*dominated samples, compared with *L. crispatus-*dominated samples. The difference became more pronounced when only women with multiple HPV and multiple oncogenic HPV types were included in the analysis (OR = 2.5, 95% CI 1.1–5.9 for multiple HPV infection; OR = 2.4, 95% CI 1.0–5.8 for multiple oncogenic HPV infection; Table 1 and Supplementary Table 3).

## Discussion

This is a large cross-sectional study for evaluating the relationship between vaginal microbiota and HPV infection. It is also the study carried out in a high HPV vaccine coverage country with young women, using sequencing technology. This study brings essential comparable data and a geographic contribution to the worldwide vaginal microbiome researches. Overall, a significantly higher microbiota diversity was observed in women infected with any HPV, oncogenic HPV, and multiple HPV types, than in women not infected with HPV (Table 1 and Fig. 3a, b). Further, we also demonstrated a slight but significantly increased microbiota diversity among women infected with oncogenic HPV39 and 56 (Fig. 3c). We suggest that BVABs, which have not been studied in previous HPV-related studies, together with *Sneathia*, *Prevotella*, and *Megasphaera*, are associated with HPV infection. Lastly, HPV vaccination showed a strong protective effect against the HPV types covered in the current vaccination program, without any perceptible influence on the vaginal microbiota.

Our recently-published meta-analysis showed that vaginal microbiota dominated by non-*Lactobacillus* species or *L. iners* have a stronger association with HPV infection and dysplasia, compared with *L. crispatus*^24^. In this study, the correlation of different vaginal microbiota compositions and their relation to HPV infection was analyzed, highlighting the different infection risks posed by the various vaginal microbiota profiles (Figs. 2, 3 and Table 1). Similar to the findings in our meta-analysis, this study also showed that the non-*Lactobacillus* vaginal microbiota profile was more common among women with infections due to any HPV, oncogenic HPV, and multiple HPV types (Figs. 2, 3 and Table 1). Nearly all the major species from the non-*Lactobacillus-dominant* group were found in significantly higher amounts in the HPV-infected group, compared with the HPV-uninfected group. Our data are in line with other studies and suggest that several non-*Lactobacillus* species could be used as potential biomarkers for HPV infection^12,27^. We did not find clear separation of vaginal microbiota in different HPV oncogenic potential groups, nor in HPV phylogenetic groups, which indicates a complicated interaction between HPV and vaginal microbiota.

Vaginal microbial community-state type has been used to classify the vaginal microbiota by several previous studies^14,16,17,27^. However, our study, together with other recently-published data on the vaginal microbiome, demonstrated that the majority of vaginal microbiota samples are dominated either by *L. crispatus* or *L. iners*, and individuals with a low-*Lactobacillus* vaginal microbial community, which are commonly colonized by bacteria such as *Gardnerella*, *Prevotella*, *Sneathia*, *and* BVABs^17,18,28^. Considering the proportion of low-*Lactobacillus* cases, more detailed community state types of bacteria other than *Lactobacilli* may be useful for vaginal microbiome research. Notably, in our study, we collected all published BVAB-related sequences and identified them from the ASVs, while the identifications were validated with BVAB qPCR primers. The total prevalence of BVAB in our study is comparable to those found in the human microbiome project^11,18^. The high prevalence of BVAB 1 in HPV-positive women indicates a potential bacterial vaginosis condition in HPV-infected young women and probably a non-vaginosis condition in HPV-uninfected young women with the non-*Lactobacillus* dominated vaginal microbiota. In addition, similar to other studies, we found a strong relationship between HPV infection and enrichment of bacteria, including *Sneathia*, which correlates with cervical neoplasm, and *Prevotella*, which contributes to HPV persistent infection^25,29,30^.

Vaccination status showed no influence on vaginal microbiota. However, this study revealed its strong protection against HPV infection, especially against infection with multiple HPV types, demonstrating the success of the Swedish national HPV vaccine program (Table 1 and Supplementary Table 3)^7–9^. Nevertheless, we found that women in the age group 19–24 had the highest risk of being infected with any HPV, oncogenic HPV, or multiple HPV types (Table 1 and Supplementary Table 3). The high HPV infection rate is mainly caused by HPV types that are not covered in the vaccine^7^. So, although the vaccination coverage is over 80% among the youth clinic participants, the HPV infection rate in this group is still high. Another possible reason is probably due to nearly all the samples from women below 23 being from the youth clinic, frequented by women seeking advice on birth control or sexually transmitted diseases^7–9^. This analysis may therefore be considered biased towards sexually active young women with possible symptoms of sexually transmitted diseases. This is also supported by the significantly higher HPV prevalence among the youth clinic participants than the older cervical screening participants (Supplementary Table 1). Notably, although sample source, age, and vaccination status affected the HPV infection status (Figs. 1, 5 and Supplementary Figs. 3 and 4), these factors did not seem to contribute to variability in the vaginal microbiota. This ruled out the confounders for microbiota analysis related to HPV infection and suggested that the relationship between HPV infection and vaginal microbiota is solid (Table 1, Fig. 2 and Supplementary Figs. 3 and 4).

Current studies on the association between the vaginal microbiota and HPV infection are mainly carried out during the reproductive age. However, young women, especially teenagers, constitute a high-risk group for HPV infection and studies have shown a peak incidence at 21 years of age^7^. Vaginal microbiota and related changes at an early stage of life may have more fundamental effects during later life, such as oncogenic risk. Our study is a large cross-sectional study of vaginal microbiota using the sequencing method focused on young women (14–29 years). Although our study lacks clinical data such as vaginal pH or data on vaginal infections other than HPV, compared to other vaginal microbiota studies, our study design limits the effect from confounders such as the variability of hormone and immunity levels due to a large age span. Further, this young group gives us a unique group with high HPV infection prevalence^7^. The samples from cervical screening, aged 23 years and above, have an HPV prevalence of 34.1% (Supplementary Table 1). The two sample sources are therefore combined to obtain a balanced HPV infection cohort, to study the association between HPV infection and vaginal microbiota. Nevertheless, most of the participants in this study are Caucasian women. This allows us to limit confounders by racioethnic diversity that may affect vaginal microbiome^18^.

Studies have indicated that some of *Lactobacillus* species, such as *L. gasseri*, may potentially be beneficial for HPV clearance^14,25^. Recent findings suggest a possible association between certain compositions of vaginal microbiota and HPV clearance or progression to cervical dysplasia and cancer^12,24,30,31^. However, although few longitudinal studies hint about how microbiota might influence HPV persistent infection, HPV could also contribute to the change of vaginal microbiota stability and composition^26,32^. Moreover, a change in HPV infection and vaginal microbiota may occur simultaneously due to sexual activities. The potentially bi-directional effect needs to be explored further. More studies, especially longitudinal ones, are needed to help us understand which vaginal microbiota composition is related to a higher risk of HPV infection, to cervical dysplasia, and cancer development. Further, the underlying mechanisms for potential interaction between HPV and microbiota need to be deciphered.

In conclusion, this is a large cross-sectional study on young Swedish women with high HPV vaccination coverage. It demonstrates the strong association between non-*Lactobacillus*-dominated vaginal microbiota and HPV infection, including any HPV, oncogenic HPV infection, and multiple HPV types. It also indicates that HPV vaccine has little impact on vaginal microbiota, and bacterial species such as BVABs could potentially be used as a biomarker and target for HPV infection and treatment.

## Methods

### Study population

In total, 172 previously described young women and 34 new participants, between the ages of 14 and 22, who visited a youth clinic in Stockholm were included (*n* = 206)^7^. Their samples were collected during the clinical visit either by clinical staff or self-collected with a vaginal swab (FLOQSwabs™, Copan Flock Technologies, Brescia, Italy). Vaginal swab was Inserted ~2–3 cm into the vagina and swirled for ~30 s. In addition, 139 study participants between 23 and 29 years were enrolled from the population-based cervical screening at maternal health clinics (*n* = 133), and from follow-up screening at the gynecological clinic, Uppsala university hospital (*n* = 6), Uppsala, Sweden. These samples were collected by clinical staff and referred to as the cervical screening samples. Collection methods were compared prior to the study and showed no difference in microbiota composition^33^. For all the samples, the collection swabs were immediately put inside FluidX tubes (Brooks Life Sciences, Chelmsford, MA, USA) containing 0.8 ml DNA/RNA-shield (Zymo Research, Irvine, CA, USA). Information as to age, HPV vaccination status and antibiotic usage within the past 3 months was ascertained from study participants. All participation was voluntary and anonymized, and written informed consent was obtained. This study was performed according to ethical permission approved by the Stockholm Regional Ethics Committee and Uppsala Regional Ethics Committee in Sweden.

### DNA extraction

The collection fluid containing the vaginal microbiota was bead-beaten with Matrix E beads (MP Biomedicals, USA), followed by digestion with Proteinase K (20 µl of 20 mg/ml proteinase K in Proteinase K Storage Buffer for 90 min, 55 °C, 250 rpm) and purification with magnetic beads according to the manufacturer’s guidelines for the ZR-96 Genomic DNA MagPrep kit (Zymo Research, USA), as previously described^7,33^. Owing to the large sample size and long collection period, DNA extraction was conducted using two versions of the ZR-96 Genomic DNA MagPrep kits. These two versions are from product upgrades that were confirmed to produce comparable microbiota results^7,33^. In each batch for DNA extraction, we included collection fluid and Zymo Microbial Community DNA Standard (Cat. no. D6300, Zymo Research, USA) as the negative and positive extraction controls, respectively. The purified DNA was stored at −20 °C until further HPV genotyping and microbiota sequencing.

### HPV genotyping

DNA was polymerase chain reaction (PCR) amplified with broad-spectrum GP5+/6+ primers targeting the HPV L1 region, as well as HPV16 and HPV33 primers targeting the E6 region. The HPV16 E6 primers consist of HPV16E6-Forward (TCAAAAGCCACTGTGTCCTGA), HPV16E6-3-Reverse

(GCTGGGTTTCTCTACGTGTTC). HPV33 E6 primers including HPV33E6-Forward (TCGTTGGGCAGGGCGCTGTG), and HPV33E6-Reverse (CTCGTGTCCTCTCATGGCGTT) as previously described^34,35^. The PCR conditions contained initial denaturation at 94 °C for 15 min, followed by 40 cycles of denaturation at 94 °C for 30 s, annealing at 38 °C for 90 s and extension at 71 °C for 80 s, and a final extension step at 71 °C for 4 min. The PCR product was further evaluated using a multiplex bead-based assay on a MAGPIX instrument (Luminex Inc., USA), as reported previously^4,7^. Probes for detecting HPV16 E6p (GTCTTGTTGCAGATCATCAAG) and HPV33E6p (GAAACTGCACTGTGACGTGTA) were added^34,35^. Twenty-seven types of HPV were analyzed, including fifteen oncogenic HPVs (HPV16, 18, 31, 33, 35, 39, 45, 51, 52, 56, 58, 59, 68, 73, 82), six probably oncogenic HPVs (HPV26, 30, 53, 66, 67, 69), and six non-oncogenic HPVs (HPV6, 11, 42, 43, 44, 70)^4,7^.

### Microbiota sequencing

The V3-V4 regions of the 16S rRNA genes were amplified from extracted DNA with Illumina sequencing index-binding primer pairs 341F/805R, as previously described^33^. For each batch of sample sequencing library preparation, we included DNA-free water and Zymo Microbial Community DNA Standard (Cat. no. D6305, Zymo Research, USA) as the negative and positive PCR controls, respectively. Thereafter, the 16S amplicons indexed from the samples and controls (including extraction and PCR controls) of the same batch were pooled together in equal molar amounts and subjected to an Illumina MiSeq sequencing platform (Illumina, CA, USA) with MiSeq Reagent Kit v3.

### Bioinformatics analysis

After base calling and de-multiplexing, the fastq files generated from each run were subjected to quality trimming, de-noising, merging, and chimera removal, before being processed into sequencing tables. The degenerated primers were firstly trimmed off from the paired-end reads and a preliminary quality trimming was then conducted with cutadapt (v. 2.0) with the setting of minimum *q* value of 15, maximum N base of zero, and minimum qualified read length of 120^36^. Thereafter, the processed reads were sent to DADA2 pipeline (v. 1.13.1) for further quality trimming, de-noising, merging, and chimera removal. In this step, the paired-end reads that have more than two expected errors (maxEE = c(2,2)) or derived from PhiX (rm.phix = TRUE) were discarded^37^. The low-quality tail from each read was trimmed off to keep the mean *q* value >20 (truncLen = c (265, 200)). After DADA2 de-noising, the paired-end reads were merged with at least a 30-bp overlap. Chimera checking was conducted on the merged reads and the recovered ASVs were summarized and used to generate the sequence table for the sequencing run. The sequencing content from the sequencing library controls was used to assess the performance of each sequencing run. Finally, all sequence tables of the qualified sequencing runs were parsed into a total sample sequence table, by collapsing rows of the identical ASVs from the raw sequence tables.

### Decontamination

In order to remove the ASVs from sequencing backgrounds (e.g., reagent, laboratory environment, etc.), a decontamination procedure was conducted on the merged sequence table, containing both samples and controls. As the substantial fraction of DNA from vaginal swab samples is human DNA, we employed a combined method considering both frequency and prevalence, to identify contaminant ASVs by using Decontam (v. 1.1.2) on R^38^. ASVs that show positive correlation in their reads fraction and DNA concentration in negative controls but not in samples, and ASVs that have high prevalence in controls but not in samples, were discarded. After the decontamination step, the controls were excluded from the sequence table for downstream data analysis.

### Sequence annotation

A preliminary classification was conducted in R by using DADA2 functions “assignTaxonomy” and “addSpecies” to search ASVs against the Silva database (v. 128)^39^. As *Lactobacillus* spp. are dominant in most of the vaginal microbiota, we improved the classification of the ASVs annotated as *Lactobacillus* by manually BLAST searching them in the National Center for Biotechnology Information (NCBI) database^40,41^. Moreover, bacterial vaginosis-associated bacteria (BVAB) 1, 2, and 3 were searched from ASVs containing 16S primer-binding regions in the whole genomic sequences and 16S sequences downloaded from other published works^20–22,42–48^. The classification of ASVs was further validated by searching BVAB qPCR primer sequences from the BVAB ASVs^49^. For the classification of *Lactobacillus* species and BVAB, a maximum of one mismatch was allowed from the alignment.

### Phylogenetic tree construction

To facilitate the calculation of Faith’s diversity and UniFrac distance matrix for the downstream analyses, a phylogenetic tree of the ASVs generated in this study was constructed. A multi-alignment of all ASVs was firstly conducted using MAFFT (v.7.407) with the default parameters. The phylogeny was determined using FastTree2 (v.2.18). The generated tree in newick format was then loaded to FigTree for visualization and double-checking whether the phylogenies of the ASVs correspond to their taxonomic annotations.

### Alpha- and beta-diversity calculation

The sequence table was rarefied to 4902 reads per sample, before conducting a diversity calculation in R by using the package “vegan” (v. 2.5-6). Alpha diversities (i.e., Shannon, Chao 1 and Faith’s phylogenetic diversity) were calculated using the functions from the same package, which were visualized in violin plots using the package “ggplot2” (v. 3.2.1). PCoA was conducted based on Bray-Curtis distance matrix and weighted UniFrac distance matrix, using the package “phyloseq” (v. 1.26.1).

### Statistics

To test the differences among microbial alpha diversities, the Wilcoxon rank-sum test and Kruskal–Wallis test were employed when comparing diversities between two groups and more than two groups, respectively. Benjamini–Hochberg correction was conducted to decrease the false discovery rate for multiple tests. The same test method was also conducted to identify taxa that are significantly different in proportional abundance between the cohorts. The association between microbiota community structure and HPV, oncogenic HPV, or multiple HPV types was analyzed by multivariable logistic regression, adjusted for age, HPV vaccination status, sample source, and presented as OR and 95% CI, with 95% CI not including OR = 1, indicating statistically significant differences. The relationship among HPV infection, vaginal microbiota, age, sample source, and vaccination status were compared by Fisher’s exact test.

### Reporting summary

Further information on research design is available in the Nature Research Reporting Summary linked to this article.

## Supplementary information

Supplementary Information

Reporting Summary

## Data Availability

All data generated or analyzed in this study are included in this published article (and the Supplementary Information). The sequencing reads have been submitted to the European Nucleotide Archive (ENA) under accession number PRJEB34755. To download the BVAB sequences and the *Lactobacillus* species sequences generated in this study, please go to https://github.com/ctmrbio/BVAB-and-Lac-sequences.

## Code availability

The code can be accessed upon request.
