# Supplementary material for: Vaginal microbiota and human papillomavirus infection among young Swedish women
Source: NPJ Biofilms Microbiomes. 2020 Oct 12;6:39. doi: 10.1038/s41522-020-00146-8 (PMC7552401; doi:10.1038/s41522-020-00146-8)
Supplement: Supplementary file 1 — Supplementary Information [file 41522_2020_146_MOESM1_ESM.pdf]

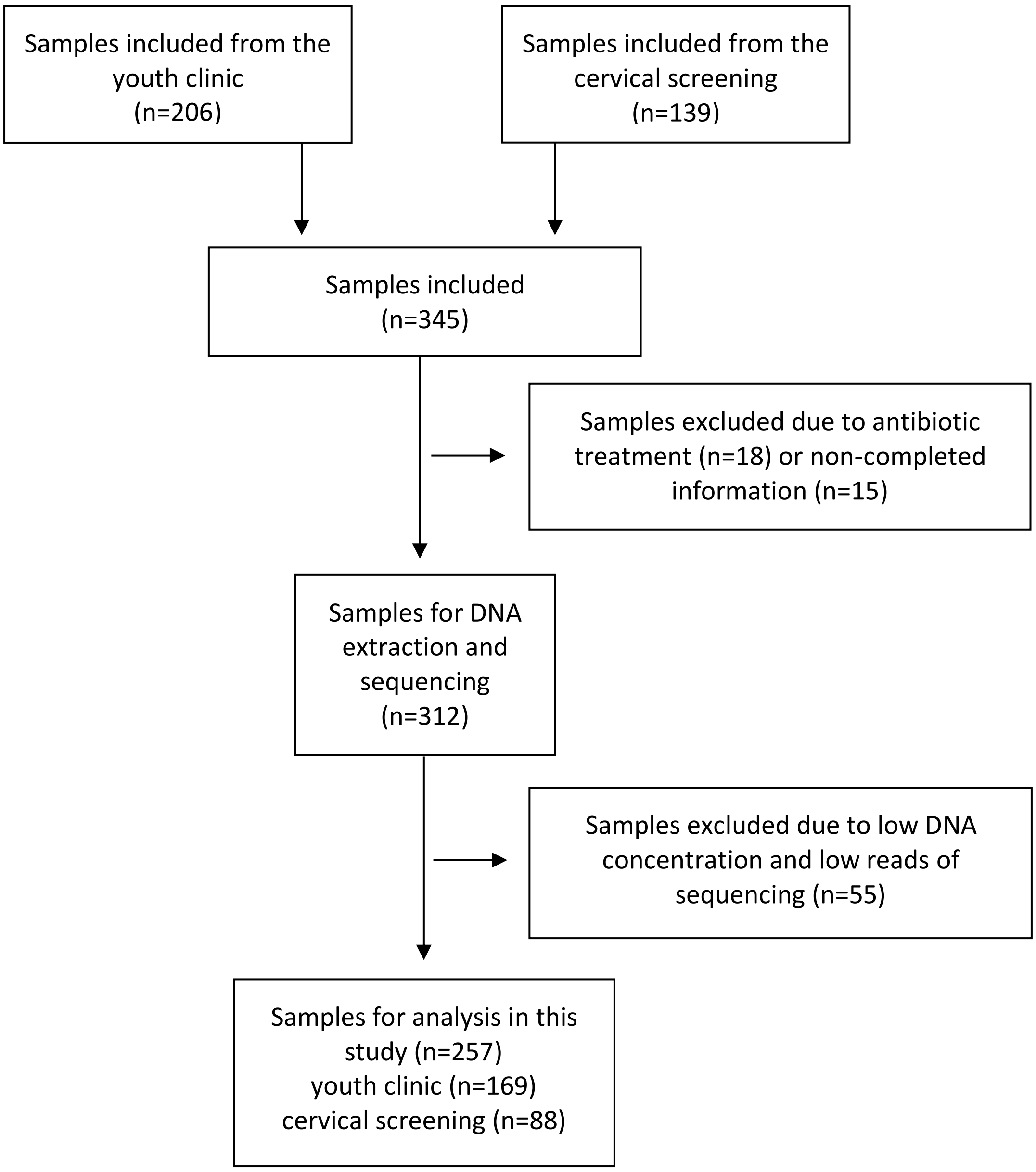

Supplementary Figure 1. Flowchart of samples included in the HPV and microbiota study.

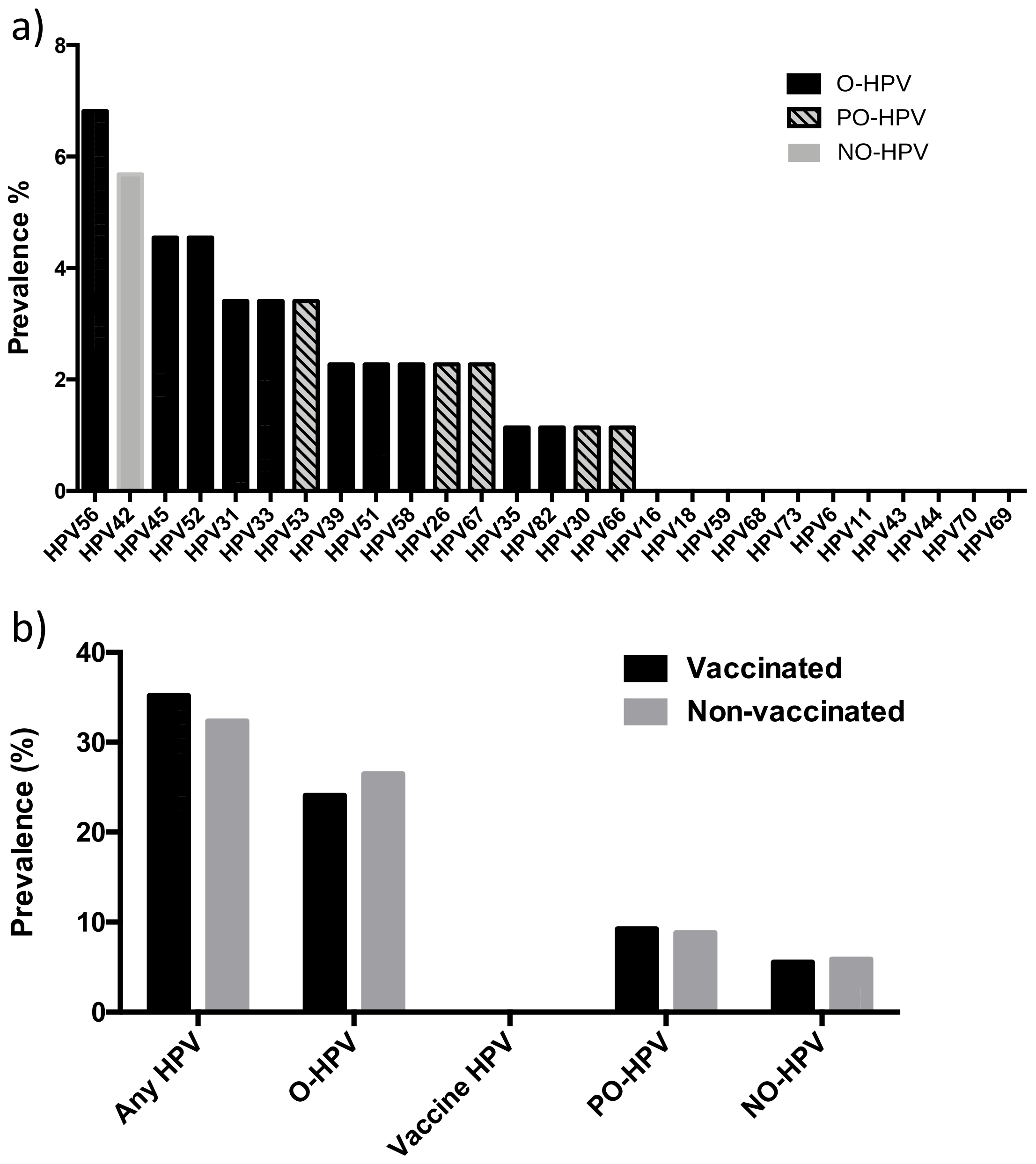

Supplementary Figure 2. HPV infection status among women in the cervical screening cohort. **a)** HPV prevalence of 27 HPV types in the cervical screening samples. The top oncogenic HPVs are HPV56, 45, 52, 31 and 33, and no HPV6, 11, 16 and 18 (HPV types covered in vaccine program) were observed. **b)** Prevalence comparison of HPV infection, oncogenic HPVs, HPV covered in vaccine, probably oncogenic HPVs and non-oncogenic HPVs among vaccinated and non-vaccinated young women. None of the HPV infection groups showed significant difference between vaccinated and non-vaccinated women. Statistical significance between the vaccinated groups and non-vaccinated groups was analysed by Fisher's exact test ($p>0.05$). (O-HPV: oncogenic HPV, NO-HPV: non-oncogenic HPV, PO-HPV: probably oncogenic HPV).

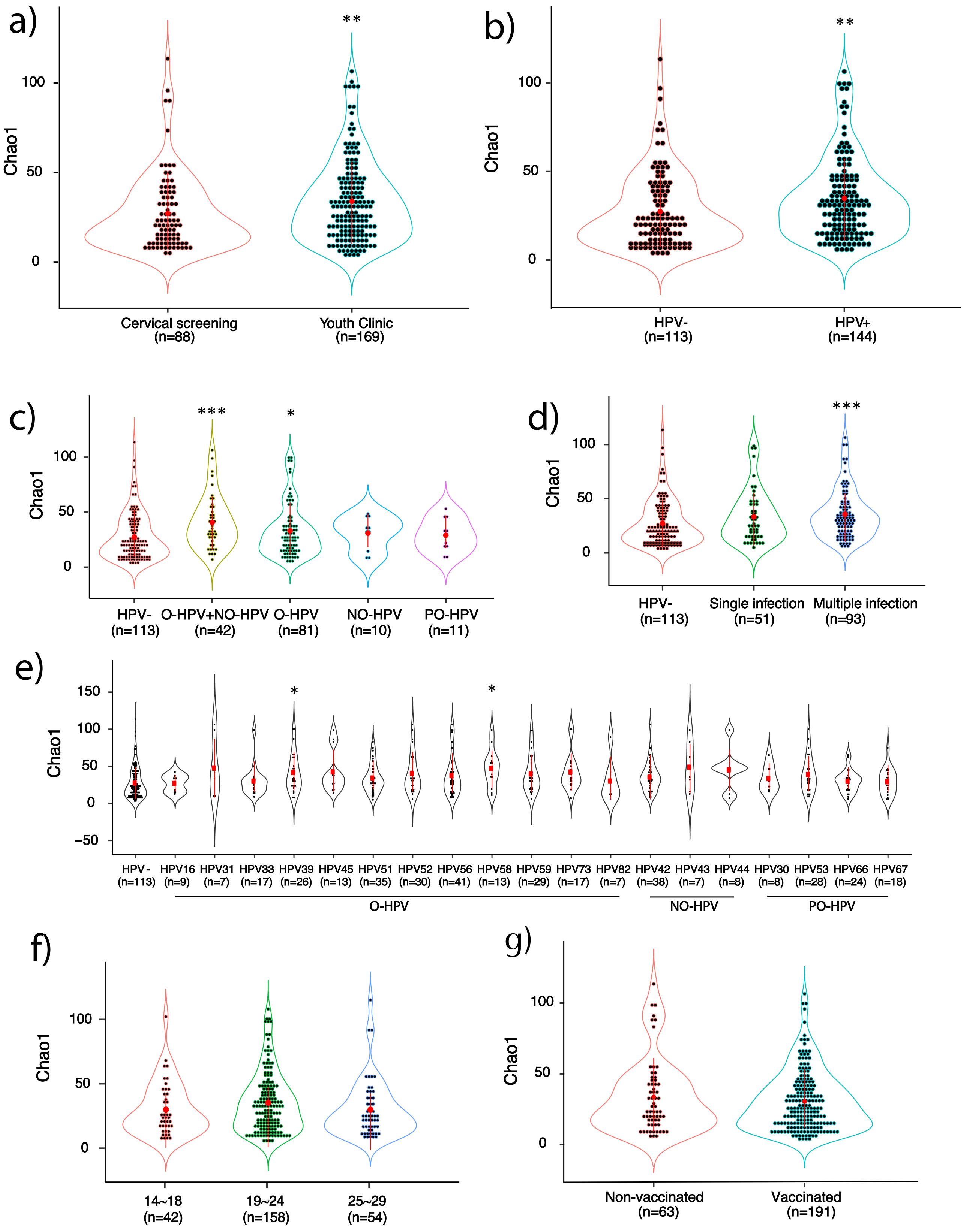

a)
Chao1
Cervical screening (n=88)
Youth Clinic (n=169)
b)
HPV- (n=113)
HPV+ (n=144)
c)
HPV- (n=113)
O-HPV+NO-HPV (n=42)
O-HPV (n=81)
NO-HPV (n=10)
PO-HPV (n=11)
d)
Single infection (n=51)
Multiple infection (n=93)
e)
HPV16 (n=9)
HPV31 (n=7)
HPV33 (n=17)
HPV39 (n=26)
HPV45 (n=13)
HPV51 (n=35)
HPV52 (n=30)
HPV56 (n=41)
HPV58 (n=13)
HPV59 (n=29)
HPV73 (n=17)
HPV82 (n=7)
HPV42 (n=38)
HPV43 (n=7)
HPV44 (n=8)
HPV30 (n=8)
HPV53 (n=28)
HPV66 (n=24)
HPV67 (n=18)
O-HPV
NO-HPV
PO-HPV
f)
14~18 (n=42)
19~24 (n=158)
25~29 (n=54)
g)
Non-vaccinated (n=63)
Vaccinated (n=191)

Supplementary Figure 3. Microbial alpha diversity based on Chao 1 analysis. **a)** Microbial diversity was significantly higher in the youth clinic samples than in the cervical screening samples (Wilcoxon one-sided test; ** $p < 0.01$). **b)** Microbial diversity was significantly higher among HPV-infected (HPV+) women than HPV-uninfected (HPV-) women (Wilcoxon one-sided test; ** $p < 0.01$). **c)** Microbial diversity of HPV-uninfected women and the women infected with oncogenic plus non-oncogenic HPVs, oncogenic HPVs, non-oncogenic HPVs, and probably oncogenic HPVs were compared. Samples of the five groups showed significantly different diversities (Kruskal-Wallis test, $p < 0.01$). Groups with oncogenic plus non-oncogenic HPVs and oncogenic HPVs showed statistical higher diversity compared to uninfected group (Wilcoxon one-sided test; * $p < 0.05$ and *** $p < 0.001$). **d)** HPV-uninfected, single-infected and multiple-infected groups had significantly different microbial diversity (Kruskal-Wallis test, $p < 0.01$). Women infected with multiple HPV types had significantly higher microbial diversity compared to uninfected women (Wilcoxon one-sided test; *** $p < 0.001$) **e)** Microbial diversity among women infected with different HPV types in comparison with uninfected women was compared. Significantly higher microbiota diversity was observed from women infected with HPV39 and 58 compared to the uninfected women (Wilcoxon one-sided test with Benjamini-Hochberg correction; * $q < 0.05$). **f)** Microbial alpha diversity analysis showed no significant difference among different age groups (Kruskal-Wallis test; $p > 0.05$). **g)** Microbial alpha diversity analysis showed no significant difference between the vaccinated and non-vaccinated groups (Wilcoxon one-sided test, $p > 0.05$). Every dot in the violin plot presented one individual. Data was presented as mean values with standard deviations. (HPV-: HPV-uninfected, HPV+: HPV-infected, O-HPV: oncogenic HPV, NO-HPV: non-oncogenic HPV, PO-HPV: probably oncogenic HPV).

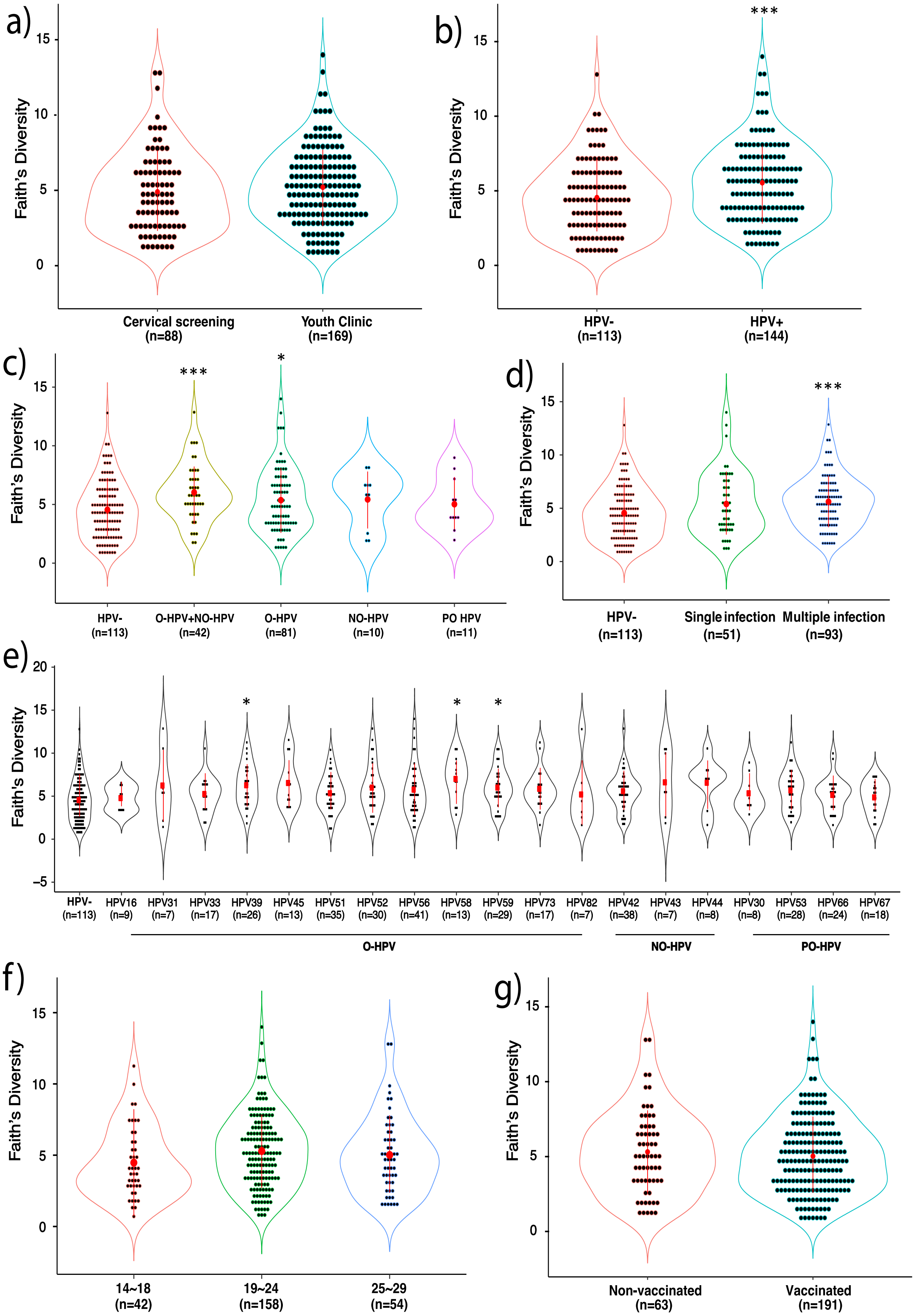
a)
Faith's Diversity
Cervical screening (n=88)
Youth Clinic (n=169)
b)
Faith's Diversity
***
HPV- (n=113)
HPV+ (n=144)
c)
Faith's Diversity
***
*
HPV- (n=113)
O-HPV+NO-HPV (n=42)
O-HPV (n=81)
NO-HPV (n=10)
PO HPV (n=11)
d)
Faith's Diversity
***
HPV- (n=113)
Single infection (n=51)
Multiple infection (n=93)
e)
Faith's Diversity
HPV- (n=113)
HPV16 (n=9)
HPV31 (n=7)
HPV33 (n=17)
HPV39 (n=26)
HPV45 (n=13)
HPV51 (n=35)
HPV52 (n=30)
HPV56 (n=41)
HPV58 (n=13)
HPV59 (n=29)
HPV73 (n=17)
HPV82 (n=7)
HPV42 (n=38)
HPV43 (n=7)
HPV44 (n=8)
HPV30 (n=8)
HPV53 (n=28)
HPV66 (n=24)
HPV67 (n=18)
O-HPV
NO-HPV
PO-HPV
f)
Faith's Diversity
14~18 (n=42)
19~24 (n=158)
25~29 (n=54)
g)
Faith's Diversity
Non-vaccinated (n=63)
Vaccinated (n=191)

Supplementary Figure 4. Microbial alpha diversity analysis based on Faith's phylogenetic diversity. **a)** Microbial diversity comparison showed no difference between the youth clinic and the cervical screening samples (Wilcoxon one-sided test; $p > 0.05$) **b)** Microbial diversity was significantly higher among HPV-infected women than HPV-uninfected women (Wilcoxon one-sided test; *** $p < 0.001$) **c)** Microbial diversity of HPV-uninfected women and the women infected with oncogenic plus non-oncogenic HPVs, oncogenic HPVs, non-oncogenic HPVs, and probably oncogenic HPVs were compared. Samples of the five groups showed significantly different diversities (Kruskal-Wallis test, $p < 0.01$). Groups with oncogenic plus non-oncogenic HPVs and oncogenic HPVs showed statistical higher diversity compared to HPV-uninfected group (Wilcoxon one-sided test; * $p < 0.05$ and *** $p < 0.001$). **d)** HPV-uninfected, single-infected and multiple-infected groups have significantly different microbial diversity (Kruskal-Wallis test, $p < 0.01$). Women infected with multiple HPV types was found having significantly higher microbial diversity compared to uninfected women. (Wilcoxon one-sided test; *** $p < 0.001$). **e)** Microbial diversity among women infected with different HPV types in comparison with uninfected women. Significantly higher microbiota diversity was demonstrated from women infected with HPV39, 58 and 59 compared to uninfected women (Wilcoxon one-sided test adjusted by Benjamini-Hochberg correction; * $q < 0.05$). **f)** Microbial alpha diversity analysis showed no difference among different age groups (Kruskal-Wallis test; $p > 0.05$). **g)** Microbial alpha diversity analysis did not show difference in vaccinated and non-vaccinated groups (Wilcoxon one-sided test; $p > 0.05$). Data was presented as mean values with standard deviations. (HPV-: HPV-uninfected, HPV+: HPV-infected, O-HPV: oncogenic HPV, NO-HPV: non-oncogenic HPV, PO-HPV: probably oncogenic HPV).

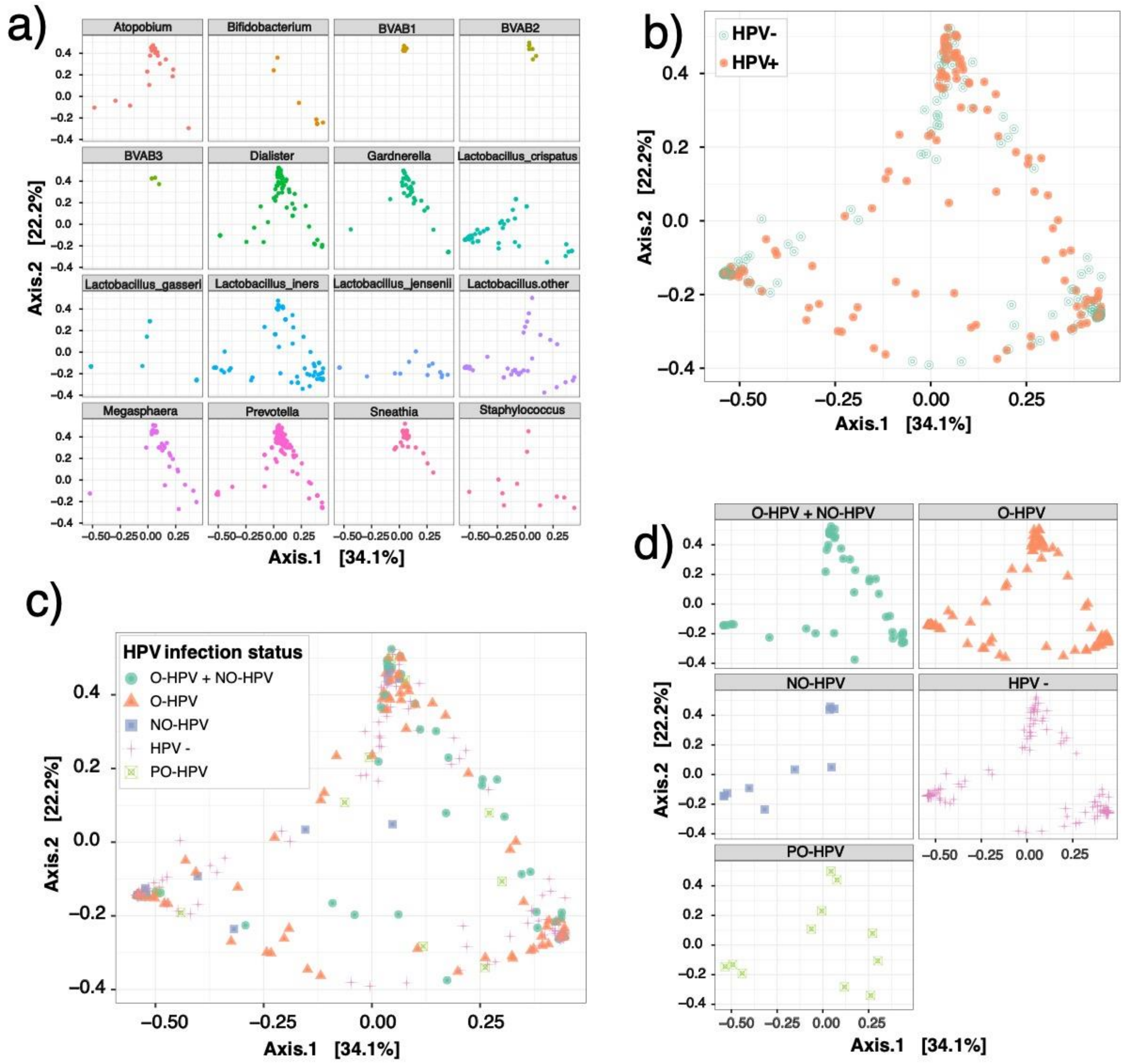

Supplementary Figure 5. Principal coordinates analysis (PCoA) of microbiota data based on Bray-Curtis distance. **a)** The contributions of the amplicon sequence variants (ASVs) from the major genera and species in the PCoA plot of main Figure 1d. The demonstrated genera and species are the same as main Figure 2a and 2b, which adopt the following cut off for taxa selection: 1) all BVABs. 2) Bacteria with over 1% mean relative abundance in all the samples. 3) *Lactobacillus* species that have more than 10% of reads in any sample. 4) Non-*Lactobacillus* genera that have over 30% of reads in any sample**. b)** PCoA plot with the samples colored according to HPV infectious status. No clear separation was observed between HPV-uninfected and HPV infected samples. **c)** No clear separation was observed in the PCoA plot with the samples colored according to HPV oncogenic potential. **d)** Detail PCoA presented by each HPV infection groups in **c)**. (HPV-: HPV-uninfected, HPV+: HPV-infected, O-HPV: oncogenic HPV, NO-HPV: non-oncogenic HPV, PO-HPV: probably oncogenic HPV).

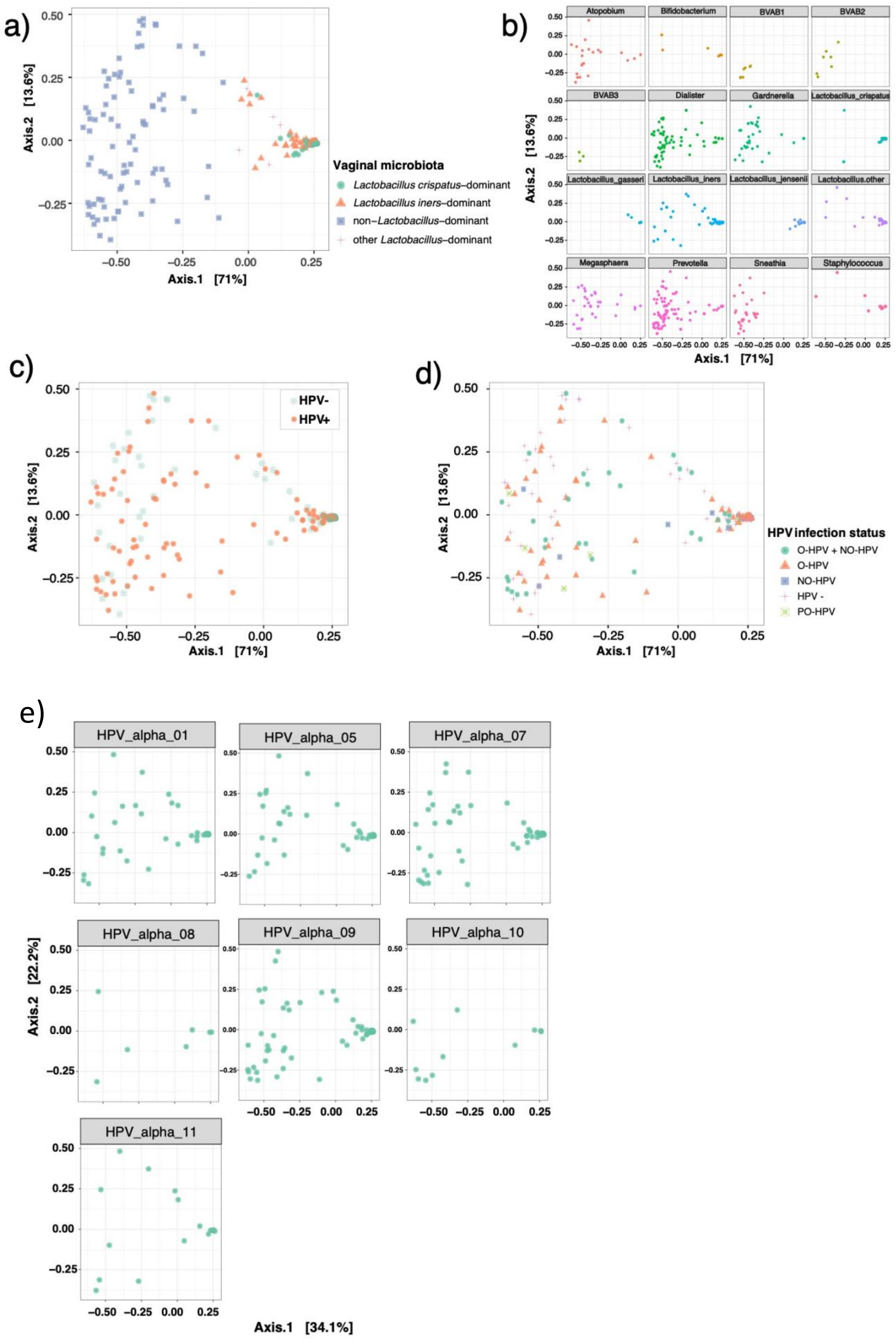
a)
Axis.1 [71%]
Axis.2 [13.6%]
Vaginal microbiota
Lactobacillus crispatus–dominant
Lactobacillus iners–dominant
non–Lactobacillus–dominant
other Lactobacillus–dominant
b)
Atopobium
Bifidobacterium
BVAB1
BVAB2
BVAB3
Dialister
Gardnerella
Lactobacillus_crispatus
Lactobacillus_gasseri
Lactobacillus_iners
Lactobacillus_jensenii
Lactobacillus.other
Megasphaera
Prevotella
Sneathia
Staphylococcus
Axis.1 [71%]
Axis.2 [13.6%]
c)
HPV-
HPV+
Axis.1 [71%]
Axis.2 [13.6%]
d)
HPV infection status
O-HPV + NO-HPV
O-HPV
NO-HPV
HPV -
PO-HPV
Axis.1 [71%]
Axis.2 [13.6%]
e)
HPV_alpha_01
HPV_alpha_05
HPV_alpha_07
HPV_alpha_08
HPV_alpha_09
HPV_alpha_10
HPV_alpha_11
Axis.1 [34.1%]
Axis.2 [22.2%]

Supplementary Figure 6. PCoA of microbiota data based on weighted UniFrac distance. **a)** PCoA plot with the samples colored according to vaginal microbial community types. Separation was seen between samples with *Lactobacillus* species and non-*Lactobacillus* species. No separation was shown between *Lactobacillus crispatus*- and *Lactobacillus iners*-dominant samples. **b)** The contributions of the ASVs from the major genera and species in the PCoA plot of **a)**. The demonstrated genera and species are the same as main Figure 2a and 2b, which adopt the following cut off for taxa selection: 1) all BVABs. 2) Bacteria with over 1% mean relative abundance in all the samples. 3) *Lactobacillus* species that have more than 10% of reads in any sample. 4) Non-*Lactobacillus* genera that have over 30% of reads in any sample**. c)** PCoA plot with the samples colored according to HPV infectious status. No clear separation was observed between HPV-uninfected and HPV infected samples. **d)** No clear separation was observed in the PCoA plot with the samples colored according to HPV oncogenic potential. **e**) PCoA plot with the samples presented separately according to HPV phylogenetic groups. (HPV-: HPV-uninfected, HPV+: HPV-infected, O-HPV: oncogenic HPV, NO-HPV: non-oncogenic HPV, PO-HPV: probably oncogenic HPV).

Supplementary Table 1. Descriptive characteristics of all women included in the study

	Cervical screening (N=88) N (%)	*Youth clinic (N=169) N (%)*	*Overall (N=257) N (%)*	*p-value (Fisher's exact test)*
Age				*<0.001*
14-18 years	-	42 (24.9)	42 (16.3)	
19-24 years	36 (40.9)	122 (72.2)	158 (61.5)	
25-29 years	52 (59.1)	2 (1.2)	54 (21.0)	
Missing	-	3 (1.8)	3 (1.2)	
HPV vaccination				*<0.001*
no	34 (38.6)	29 (17.2)	63 (24.5)	
yes	54 (61.4)	137 (81.1)	191 (74.3)	
missing	-	3 (1.8)	3 (1.2)	
HPV status				*<0.001*
No HPV	58 (65.9)	55 (32.5)	113 (44.0)	
Any HPV	30 (34.1)	114 (67.5)	144 (56.0)	
HPV types detected				
Oncogenic HPV	22 (25.0)	101 (59.8)	123 (47.9)	*<0.001*
Non-oncogenic HPV	5 (5.7)	47 (27.8)	52 (20.2)	*<0.001*
Probably oncogenic HPV	8 (9.1)	54 (32.0)	62 (24.1)	*<0.001*
HPV groups				*<0.001*
None	58 (65.9)	55 (32.5)	113 (44.0)	
Oncogenic & probably oncogenic	2 (2.3)	40 (23.7)	42 (16.3)	
Only oncogenic	20 (22.7)	61 (36.1)	81 (31.5)	
Only non-oncogenic	3 (3.4)	7 (4.1)	10 (3.9)	
Only Probably oncogenic	5 (5.7)	6 (3.6)	11 (4.3)	
Vaginal microbiota				*0.955*
L. crispatus	31 (35.2)	53 (31.4)	84 (32.7)	
L. iners	26 (29.6)	52 (30.8)	78 (30.4)	
Other Lactobacilli	3 (3.4)	6 (3.6)	9 (3.5)	
Non-Lactobacillus dominated	28 (31.8)	58 (34.3)	86 (33.5)	
Number of HPV types (any)				
Median (interquartile range)	0 (0-1)	1 (0-3)	1 (0-2)	
Maximum	3	11	11	
Number of HPV types (oncogenic)				
Median (interquartile range)	0 (0-0.5)	1 (0-2)	0 (0-2)	
Maximum	3	9	9	

Supplementary Table 2. The characteristics of all women by vaginal microbiota composition

	L.crispatus **(N=84)** *N (%)*	***L.iners*** **(N=78)** *N (%)*	**Other *Lactobacilli*** **(N=9)** *N (%)*	**Non-*Lactobacillus* dominated (N=90)** *N (%)*	**Total (N=257)** *N (%)*	***p-value (Fisher's exact test)***
HPV status						*0.043*
No HPV	41 (48.8)	41 (52.6)	3 (33.3)	28 (32.6)	113 (44.0)	
Any HPV	43 (51.2)	37 (47.4)	6 (66.7)	58 (67.4)	144 (56.0)	
HPV types detected						
Oncogenic	34 (40.5)	36 (46.2)	2 (22.2)	51 (59.3)	123 (47.9)	*0.050*
Non-oncogenic	13 (15.5)	12 (15.4)	2 (22.2)	25 (29.1)	52 (20.2)	*0.021*
Probably oncogenic	19 (22.6)	15 (19.2)	3 (33.3)	25 (29.1)	62 (24.1)	*0.105*
Age						*0.303*
14-18 years	17 (20.2)	14 (18.0)	3 (33.3)	8 (9.3)	42 (16.3)	
19-24 years	48 (57.1)	50 (64.1)	6 (66.7)	54 (62.8)	158 (61.5)	
25-29 years	18 (21.4)	14 (18.0)	0 (0.0)	22 (25.6)	54 (21.0)	
missing	1 (1.2)	0 (0.0)	0 (0.0)	2 (2.3)	3 (1.2)	
HPV vaccination						*0.693*
no	18 (21.4)	21 (26.9)	1 (11.1)	23 (26.7)	63 (24.5)	
yes	64 (76.2)	56 (71.8)	8 (88.9)	63 (73.3)	191 (74.3)	
missing	2 (2.4)	1 (1.3)	0 (0.0)	0 (0.0)	3 (1.2)	
Sample source						*0.955*
Screening cohort	31 (36.9)	26 (33.3)	3 (33.3)	28 (32.6)	88 (34.2)	
Youth clinic	53 (63.1)	52 (66.7)	6 (66.7)	58 (67.4)	169 (65.8)	

Supplementary Table 3. The characteristics of all included women by HPV status

	Overall (N=257) *N (%)*	*HPV- (N=113)* *N (%)*	*HPV+ (N=144)* *N (%)*	*p-value (Fisher test)*	*Oncogenic HPV (N=126)* *N (%)*	*p-value (Fisher test)* (compared to HPV-)	*Multiple HPV (N=71)* *N (%)*	*p-value (Fisher's exact test)* (compared to HPV-)
**Age**				*<0.001*		*<0.001*		*<0.001*
14-18 years	42 (16.3)	24 (21.2)	18 (12.5)		15 (12.2)		8 (11.8)	
19-24 years	158 (61.5)	52 (46.0)	106 (73.6)		92 (74.8)		55 (80.9)	
25-29 years	54 (21.0)	36 (31.9)	18 (12.5)		14 (11.4)		4 (5.9)	
missing	3 (1.2)	1 (0.9)	2 (1.4)		2 (1.6)		1 (1.5)	
**Sample source**				*<0.001*		*<0.001*		*<0.001*
Youth clinic	169 (65.8)	55 (48.7)	114 (79.2)		104 (82.5)		63 (92.7)	
Cervical screening	88 (34.2)	58 (51.3)	30 (20.8)		22 (17.5)		5 (7.4)	
**HPV vaccination**				*0.884*		*0.766*		*<0.001*
no	63 (24.5)	27 (23.9)	36 (25.0)		33 (26.2)		51 (75.0)	
yes	191 (74.3)	84 (74.3)	107 (74.3)		92 (73.0)		17 (25.0)	
missing	3 (1.2)	2 (1.8)	1 (0.7)		1 (0.8)		-	
**Vaginal microbiota**				*0.043*		*0.050*		*0.052*
L. crispatus	84 (32.7)	41 (36.3)	43 (29.9)		34 (27.6)		19 (27.9)	
L. iners	78 (30.4)	41 (36.3)	37 (25.7)		36 (29.3)		17 (25.0)	
Other Lactobacilli	9 (3.5)	3 (2.7))	6 (4.2)		2 (1.6)		2 (2.9)	
Non-Lactobacillus dominated	86 (33.5)	28 (24.8)	58 (40.3)		51 (41.5)		30 (44.1)	
