# Supplementary material for: Vaginal microbiota and human papillomavirus infection among young Swedish women
Source: NPJ Biofilms Microbiomes. 2020 Oct 12;6:39. doi: 10.1038/s41522-020-00146-8 (PMC7552401; doi:10.1038/s41522-020-00146-8)
Supplement: Supplementary file 2 — Reporting Summary [file 41522_2020_146_MOESM2_ESM.pdf]

nature research

Corresponding author(s): Juan Du

Last updated by author(s): Jul 1, 2020

Reporting Summary

Nature Research wishes to improve the reproducibility of the work that we publish. This form provides structure for consistency and transparency in reporting. For further information on Nature Research policies, see our Editorial Policies and the Editorial Policy Checklist.

Statistics

For all statistical analyses, confirm that the following items are present in the figure legend, table legend, main text, or Methods section.

n/a	Confirmed	
☐	☒	The exact sample size (*n*) for each experimental group/condition, given as a discrete number and unit of measurement
☐	☒	A statement on whether measurements were taken from distinct samples or whether the same sample was measured repeatedly
☐	☒	The statistical test(s) used AND whether they are one- or two-sided<br>*Only common tests should be described solely by name; describe more complex techniques in the Methods section.*
☐	☒	A description of all covariates tested
☐	☒	A description of any assumptions or corrections, such as tests of normality and adjustment for multiple comparisons
☐	☒	A full description of the statistical parameters including central tendency (e.g. means) or other basic estimates (e.g. regression coefficient) AND variation (e.g. standard deviation) or associated estimates of uncertainty (e.g. confidence intervals)
☐	☒	For null hypothesis testing, the test statistic (e.g. *F*, *t*, *r*) with confidence intervals, effect sizes, degrees of freedom and *P* value noted<br>*Give P values as exact values whenever suitable.*
☒	☐	For Bayesian analysis, information on the choice of priors and Markov chain Monte Carlo settings
☒	☐	For hierarchical and complex designs, identification of the appropriate level for tests and full reporting of outcomes
☒	☐	Estimates of effect sizes (e.g. Cohen's *d*, Pearson's *r*), indicating how they were calculated

Our web collection on statistics for biologists contains articles on many of the points above.

Software and code

Policy information about availability of computer code

Data collection	The HPV PCR product was evaluated using a multiplex bead-based assay on a MAGPIX instrument (Luminex Inc., USA). The16S amplicons indexed from the samples and controls were subjected to an Illumina MiSeq sequencing platform (Illumina, CA, USA) with MiSeq Reagent Kit v3.
Data analysis	A preliminary quality trimming was then conducted with cutadapt (v. 2.0), then the processed reads were sent to a DADA2 pipeline (v. 1.13.1) for further quality trimming, de-noising, merging and chimera removal. Thereafter, a combined method considering both frequency and prevalence, to identify contaminant amplicon sequence variants (ASVs) by using Decontam (v. 1.1.2) on R. A preliminary classification was conducted in R by using DADA2 functions “assignTaxonomy” and “addSpecies” to search ASVs against the Silva database (v. 128) .<br>Diversity calculation in R by using the package “vegan” (v. 2.5-6). Alpha diversities (i.e., Chao 1, Shannon and Faith’s phylogenetic diversity) were calculated using the functions from the same package, which were visualised in violin plots using the package “ggplot2” (v. 3.2.1). Principal coordinates analysis (PCoA) was conducted based on weighted UniFrac distance matrix, using the package “phyloseq” (v. 1.26.1).

For manuscripts utilizing custom algorithms or software that are central to the research but not yet described in published literature, software must be made available to editors and reviewers. We strongly encourage code deposition in a community repository (e.g. GitHub). See the Nature Research guidelines for submitting code & software for further information.

Data

Policy information about availability of data

All manuscripts must include a data availability statement. This statement should provide the following information, where applicable:

- Accession codes, unique identifiers, or web links for publicly available datasets
- A list of figures that have associated raw data
- A description of any restrictions on data availability

All data generated or analysed during this study are included in this published article (and the Supplementary Information). The sequencing reads have been submitted to the European Nucleotide Archive (ENA) under accession number PRJEB34755. To download BVAB sequences used in this study, and the Lactobacillus species sequences generated in this study, please go to https://github.com/ctmrbio/BVAB-and-Lac-sequences .

Field-specific reporting

Please select the one below that is the best fit for your research. If you are not sure, read the appropriate sections before making your selection.

- [x] Life sciences
- [ ] Behavioural & social sciences
- [ ] Ecological, evolutionary & environmental sciences

For a reference copy of the document with all sections, see nature.com/documents/nr-reporting-summary-flat.pdf

Life sciences study design

All studies must disclose on these points even when the disclosure is negative.

Sample size	We carried out a cross-sectional study with 345 young Swedish women. Before sample collection, we used "epitools" with power 0.8 and significant level 0.05, and sample Sizes Tool for Case-Control Microbiome Studies (https://fedematt.shinyapps.io/shinyMB/) with power 0.95. Both tools suggested around 100 samples in each group, which means around 200 samples in total. Our sample size is above this number and should provide enough power to perform a solid statistic analysis.
Data exclusions	A total of 33 women were excluded from the study due to antibiotics usage within the past three months (n = 18) or incomplete clinical information (n = 15). Samples with low DNA concentration and low reads in sequencing (n = 55) were excluded from downstream microbiota analysis.
Replication	There is only one sample from each participate and no replication was able to obtained during this study.
Randomization	All participates were randomly collected without selection both from youth clinic and cervical screening clinics.
Blinding	Investigators were blinded to group allocation during sample collection and HPV typing. After HPV typing, microbiota analysis is based on HPV data without blinding. This is due to this study design is to find the relationship between HPV and microbiota. One of the two factors need to be fixed for dividing groups.

Reporting for specific materials, systems and methods

We require information from authors about some types of materials, experimental systems and methods used in many studies. Here, indicate whether each material, system or method listed is relevant to your study. If you are not sure if a list item applies to your research, read the appropriate section before selecting a response.

Materials & experimental systems

n/a	Involved in the study	
x		Antibodies
x		Eukaryotic cell lines
x		Palaeontology and archaeology
x		Animals and other organisms
	x	Human research participants
x		Clinical data
x		Dual use research of concern

Methods

n/a	Involved in the study	
x		ChIP-seq
x		Flow cytometry
x		MRI-based neuroimaging

Human research participants

Policy information about studies involving human research participants

Population characteristics	In total, 172 previously described young women and 34 new participants, between the ages of 14 and 22, who visited a youth clinic in Stockholm were included (n = 206). In addition, 139 study participants between 23 and 29 years were enrolled from the population-based cervical screening at maternal health clinics (n=133), and from follow-up screening at the gynecological clinic, Uppsala university hospital (n=6), Uppsala, Sweden. Information as to age, HPV vaccination status and antibiotic usage within the past three months was ascertained from study participants. All participation was voluntary and anonymised, and written informed consent was obtained.
Recruitment	Youth clinic samples were collected during the clinical visit either by clinical staff or self-collected with a vaginal swab (FLOQSwabs™, Copan Flock Technologies, Brescia, Italy) inserting it around 2-3 cm into the vagina and swirling it for around 30 seconds. Cervical screening samples were collected by clinical staff. Collection methods were compared prior to the study and showed no difference in microbiota composition.
Ethics oversight	This study was performed according to ethical permission approved by the Stockholm Regional Ethics Committee and Uppsala Regional Ethics Committee.

Note that full information on the approval of the study protocol must also be provided in the manuscript.
